# Anti-Mullerian hormone concentrations in individual follicular fluids within one stimulated IVF cycle resemble blood serum values

**DOI:** 10.1007/s10815-017-0908-4

**Published:** 2017-03-21

**Authors:** Michael Schenk, Julia Maria Kröpfl, Barbara Obermayer-Pietsch, Elisabeth Feldmeier, Gregor Weiss

**Affiliations:** 1 Das Kinderwunsch Institut Schenk GmbH, Am Sendergrund 11, 8143 Dobl, Austria; 2 Exercise Physiology Lab, Institute of Human Movement Sciences and Sport, ETH Zürich, Winterthurerstrasse 190, 8057 Zürich, Switzerland; 3 Division of Endocrinology and Diabetology, Department of Internal Medicine, Medical University of Graz, Auenbruggerplatz 15, 8036 Graz, Austria

**Keywords:** Anti-Mullerian hormone, Follicular fluid, IVF, Steiner-Tan needle

## Abstract

**Purpose:**

Anti-Mullerian hormone (AMH) is commonly known as the most potent marker for ovarian reserve due to its decline as female age increases. While serum AMH (sAMH) levels have been intensively investigated, there is less data regarding AMH concentrations in follicular fluid (FF), since FF has usually been designated as waste product during oocyte collection in assisted reproductive technologies. This pilot study investigated follicle AMH concentrations (fAMH) of several follicles per ovary, individually collected with the Steiner-Tan needle, and compared them to sAMH concentrations in women undergoing IVF treatment. We hypothesized that there is no difference of fAMH concentrations in individual follicles and that these concentrations resemble the sAMH value of the patient.

**Methods:**

Patients were stimulated with a gonadotropin-releasing hormone antagonist ovarian hyperstimulation protocol. On the day of oocyte retrieval, serum samples and FF from all individual follicles from one stimulated IVF cycle were collected and individually analyzed for AMH concentrations.

**Results:**

Intracyclic mean fAMH values (*n*_follicle_ = 2–14) were significantly correlated to sAHM values (*ρ* = 0.85, *p* < 0.001) and showed a trend to be negatively associated with age (*ρ* = −0.43, *p* = 0.06). Mean intrapatient fAMH concentrations differed significantly (*p* < 0.001). Furthermore, significant correlations of sAMH with individual fAMH values of the first five follicles of each patient were observed.

**Conclusions:**

In conclusion, our results clearly showed that individual fAMH concentrations reflected sAMH values and that fAMH concentrations did not significantly differ within one patient. In future studies, it will be interesting to correlate individual fAMH values to the respective embryo development and overall pregnancy outcome in order to improve IVF treatments and to refrain from embryo overproduction.

## Introduction

Follicular fluid (FF) is a mixture of chemical constituents, comprising a variety of different proteins as well as growth factors, reactive oxygen species, anti-apoptotic factors, fatty acids, sugars, and hormones [1]. Among them, the Anti-Mullerian hormone (AMH), a homodimeric glycoprotein which belongs to the transforming growth factor-β (TGF-β) family, has evolved to become one of the most prominent targets for reproductive health research [2]. Anti-Mullerian hormone is secreted primarily by granulosa cells of ovarian follicles during early stages of follicle development (pre-antral and antral follicles) [3] but has also been found in endometrial and endometriotic tissue [4]. Females are born with a defined number of primordial follicles, whose quantity and quality define the ovarian reserve [5]. Oocytes within primordial follicle rest in a dormant state of meiosis I until puberty, and until then, the granulosa cells do not secrete AMH. A recent study demonstrated the beginning of AMH secretion with the recruitment of the follicles [6], with maximum AMH values at the age of 15.8 years. After a plateau phase until the age of 25 years, AMH concentrations start to decrease and inversely correlate with age. This emphasizes AMH as the most potent marker for ovarian reserve in women of 25 years and older [7] among other parameters commonly used such as antral follicle count, follicle stimulating hormone (FSH), luteinizing hormone (LH), estradiol, or inhibin B. During the menstrual cycle serum AMH (sAMH), values remain relatively stable compared to all other hormones secreted by the ovary and can be analyzed at any day of the cycle, which is an advantage for flexible in vitro fertilization (IVF) treatment [8].

Values of sAMH have been intensively investigated [9, 10]; however, there is less data regarding the behavior of AMH concentrations in FF (fAMH), since FF has usually been designated as waste product during oocyte collection for IVF treatment. Recent studies revealed the importance of FF in oocyte development [11], providing information on follicular growth, oocyte quality, and fertilization. However, in studies to date, FFs have either been pooled [12] or solely the dominant follicle was examined [13]. Information on individual fAMH concentrations within one stimulated IVF cycle is still not available, although it is tempting to speculate that individual fAMH could be a potential predictor of fertilization success in IVF treatments, since it had already been found to correlate with the respective embryo and IVF outcome, at least regarding the FF of the dominant follicle [14]. Due to different study designs and methods used (dominant or pooled follicle analysis), it is hardly possible to compare results and draw any conclusions. Comparing all individual fAMH values with the respective embryo and IVF outcome, however, has not yet been done and would emphasize fAMH even more as potent biomarker in IVF treatment by facilitating and complementing the embryo selection process.

In this pilot study, we demonstrated that all mature follicles during a stimulated IVF cycle could be aspirated individually with the Steiner-Tan needle [15] for fAMH analysis. We hypothesized that there was no difference between fAMH concentrations of individual follicles within one patient and that these concentrations resembled the respective sAMH value of the patient.

## Material and methods

### Study design

Blood serum (*n* = 17) and follicular fluid samples (*n* = 20) of female volunteers, age 26–43, undergoing IVF treatment were prospectively collected and analyzed for AMH concentrations if they met the following criteria: (1) both ovaries present, (2) BMI between 19 and 30, (3) adequate visualization of ovaries in transvaginal ultrasound scan, (4) written informed consent of the patients, and (5) stimulation with gonadotropin-releasing hormone (GnRH) antagonist protocol. Samples were collected at the Kinderwunsch Institut Dobl, Austria, between 2014 and 2015. Informed consent was obtained from each woman with approval of the ethical committee of the Medical University of Graz (approval number 20–492 ex08/09).

### Ovarian hyperstimulation protocol

All women included for the study underwent GnRH antagonist protocol controlled ovarian hyperstimulation. Patients received recombinant human follicle stimulating hormone (Puregon; MSD Sharp & Dohme GMBH) for 5 days with doses according to age, weight, sAMH, and hormonal status [16, 17]. Trans-vaginal sonography was performed after 5 days and on the day of oocyte retrieval. Ultrasonographical measurement was performed using a RIC 5-9-D 4D intravaginal probe of a GE Voluson E8 BT09 ultrasound machine (both from GE Healthcare Austria GmbH). GnRH antagonist (Cetrotide, Merck KGaA) was injected to avoid premature ovulation. Triggering was initiated 35 h before the punction, administered with 5000–10,000 IU human chorionic gonadotropin (hCG) subcutaneously (Pregnyl, N.V. Organon), with dose according to body weight of the patient [16].

### Oocyte and follicular fluid retrieval

Oocyte retrieval was performed under sedation (Propofol, Fresenius Kabi Austria GmbH; Rapifen, Janssen-Cilag Pharma GmbH). Every follicle larger than 10 mm in diameter was aspirated and flushed (Flushing medium GM501 Flush; Gynemed Medizinprodukte GmbH & Co.KG) under transvaginal ultrasound guidance (GE Healthcare Austria GmbH) with a Steiner-Tan needle 17 gauge and a Steiner flush/valve (IVFETFLEX.com HandelsgmbH & Co KG) [15]. Follicles closest to the vagina were aspirated first. Follicular fluid was examined for oocytes under constant conditions of 37 °C in an IVF workstation L24E with heating stage (K-SYSTEMS Kivex Biotec A/S) and was subsequently stored at 4 °C for further hormonal analysis. The flushing volumes were examined for oocytes and discarded afterwards. The method of collection and storage of FF as well as other body liquids within the frame of IVF (blood serum, cumulus cells, seminal plasma, embryo culture supernatant) was previously described by Schenk et al. [18], providing comprehensive information on laboratory procedures and sampling techniques to enable the comparability of future studies in the field of reproductive health research.

### Blood serum collection

Blood serum was collected on day of oocyte retrieval. At least 4 ml of blood was taken by venipuncture into an 8-ml vacuette tube with serum separator (Greiner Bio-One International GmbH). Samples were centrifuged at 1800*g* for 10 min, and serum supernatant was collected and stored at 4 °C in 15-ml tubes (VWR International GmbH) for subsequent AMH analysis.

### Quantitative analysis of AMH

Serum and follicular fluid AMH concentrations were determined using electrochemiluminescence immunoassay (ECLIA) for quantitative determination (Cobas-e411 analyzer, Roche Diagnostics GmbH). The analysis is fully automated with a mean intra-assay coefficient of variability (CV) of 1.34% and a mean inter-assay CV of 3.84% for sAMH according to the manufacturer’s data sheet. Follicular fluid was centrifuged 10 min at 3000*g* before measurement. Samples were analyzed according to the manufacturer’s instructions in a measurement range of 0.01–23 ng/ml. For validation of parallelism [19] of the obtained fAMH results, samples were diluted 1:2, 1:5, 1:10, 1:20, 1:50, and 1:100 in buffer and reanalyzed.

### Statistical analysis

Data are presented as individual or mean values. All variables were tested for normal distribution with the Kolmogorov-Smirnov test. Depending on variables’ distributions, Pearson’s or Spearman’s rank correlation analysis was used to detect associations between continuous variables. An a priori power analysis revealed a necessary sample size of *n* = 17 in order to detect any significant relationship (effect size = 0.7, alpha = 0.05, 1-beta = 0.92). Differences between sAMH and intra-individual fAMH values of the first five follicles (median number of follicles in this patient cohort) were investigated by repeated measures ANOVA with Bonferroni post hoc corrections. Comparison of mean fAMH values of all follicles between patients was done by one-way ANOVA with Bonferroni post hoc corrections. A *p* value <0.05 was considered as significant.

## Results

### Patients’ characteristics

A total of 20 patients (age 35.8 ± 5.5 years; BMI 22.1 ± 2.4 kg/m^2^) undergoing IVF treatment between 2014 and 2015 were analyzed (Table 1). The number of aspirated follicles ranged from two to 14, according to the number of follicles matured during IVF treatment.

**Table 1:** Individual patient characteristics

Patients ID	Age	BMI	*n*_follicle_
1	35	23.0	5
2	32	22.3	3
3	43	20.8	3
4	30	20.3	14
5	26	21.6	6
6	32	21.3	10
7	35	21.7	5
8	41	24.6	3
9	39	19.4	12
10	42	30.1	7
11	41	21.3	5
12	30	19.9	13
13	43	20.8	6
14	36	20.3	12
15	31	21.6	4
16	33	21.8	6
17	41	24.2	8
18	43	21.2	4
19	28	22.9	2
20	35	20.3	5

*n*_*follicle*_ number of follicles collected during IVF treatment

### Inter-variable relationships

**Fig. 1:**
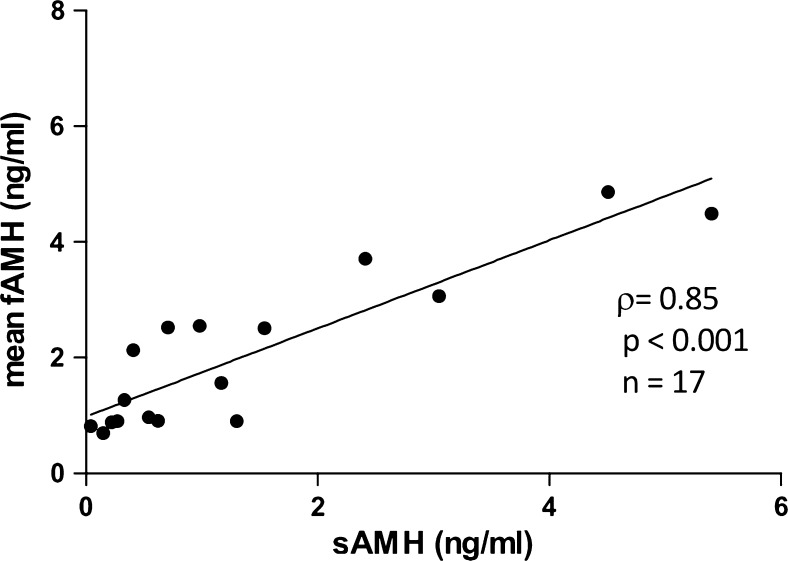
Relationship of intracyclic mean fAMH values (*n*_follikel_ = 2–14) to sAHM values (*ρ* = 0.85)

Mean fAMH values (*n*_follicle_ = 2–14) were significantly correlated to sAMH values (*ρ* = 0.85, *p* < 0.001, *n* = 17; Fig. 1). Younger patients showed by trend higher fAMH concentrations than older ones (*ρ* = −0.43, *p* = 0.06, *n* = 20), while their body mass index was not significantly associated (*p* > 0.05). Likewise, sAMH concentrations revealed a significant relationship to age (*r* = −0.53, *p* < 0.05, *n* = 17).

**Fig. 2:**
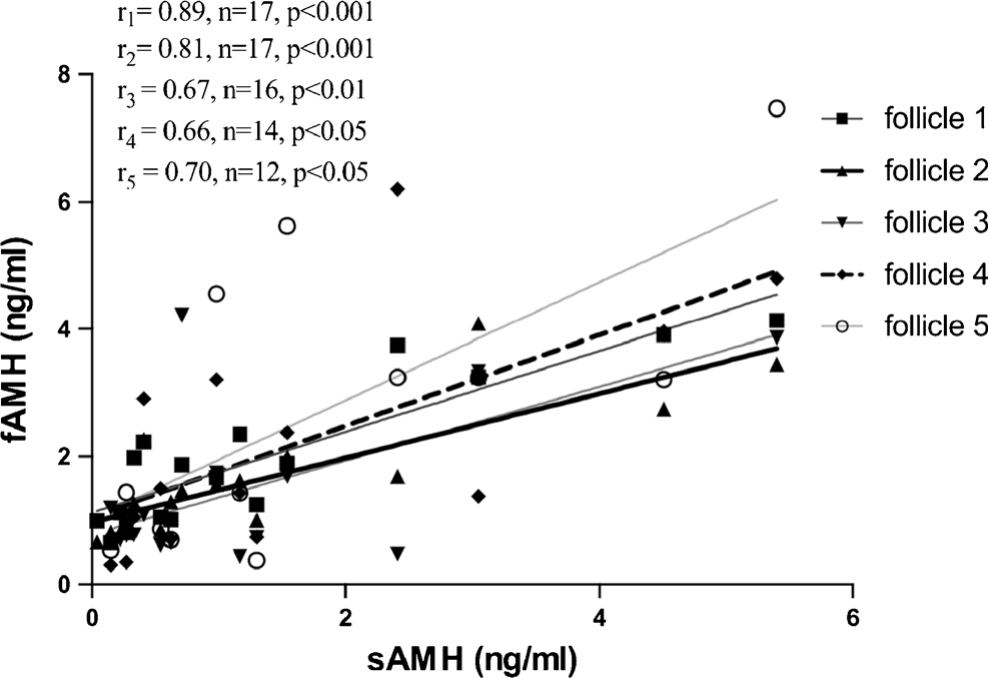
Correlation of sAMH with individual fAMH values of the first five follicles of each patient

Furthermore, significant correlations of sAMH with individual fAMH values of the first five follicles of each patient were observed (*r*_1_ = 0.89, *n* = 17, *p* < 0.001; *r*_2_ = 0.81, *n* = 17, *p* < 0.001; *r*_3_ = 0.67, *n* = 16, *p* < 0.01; *r*_4_ = 0.66, *n* = 14, *p* < 0.05; *r*_5_ = 0.70, *n* = 12, *p* < 0.05) (Fig. 2).

### Quantification of AMH concentrations in blood serum and follicular fluid: intracyclic and intrapatient analysis

It was clearly visible that AMH could be measured in an individual follicle, since AMH values among five different follicles of one patient were not significantly different from each other and comparable to sAMH values (*n* = 14, *p* > 0.05).

### Quantification of AMH concentrations in blood serum and follicular fluid: subanalyses between patients

**Fig. 3:**
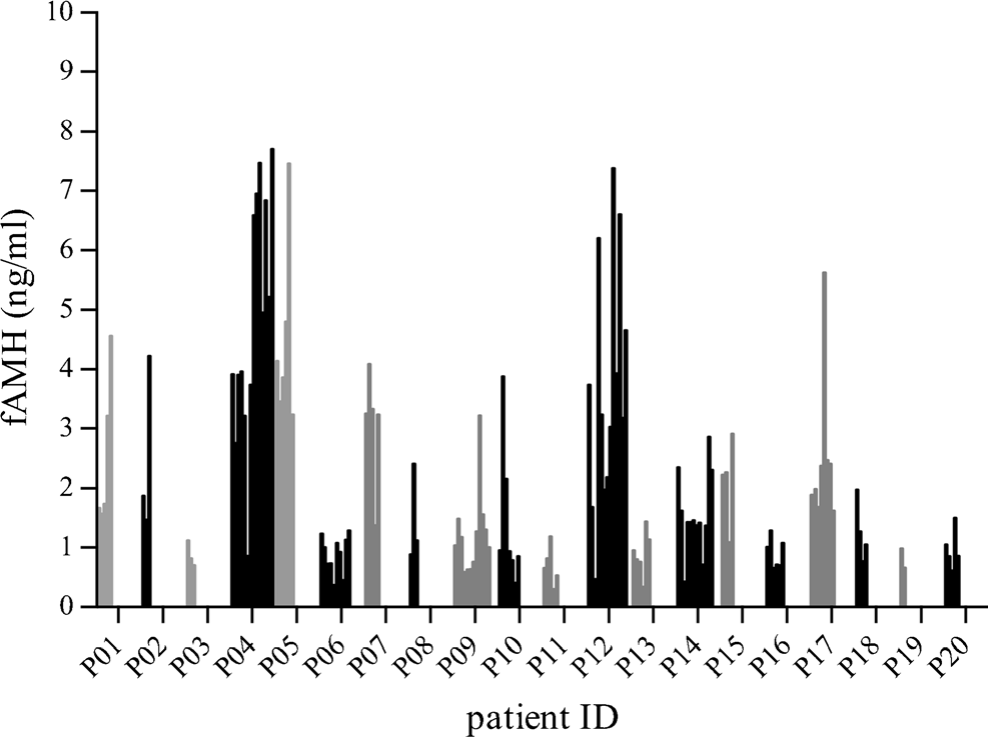
Individual follicle AMH (fAMH) values in all patients (nfollicle per patient = 2–14). Mean fAMH concentrations significantly differed between patients (one-way ANOVA, *p* < 0.001)

Mean fAMH concentrations between patients differed significantly (*p* < 0.001, *n*_follicle_ per patient = 2–14, Fig. 3).

## Discussion

In the present study, we provided evidence that fAMH concentrations differed between patients but not within one stimulated IVF cycle and that individual fAMH values correlated with sAMH data of the respective patient. We demonstrated that FF can be collected from individual follicles within one stimulated cycle (intracyclic) with the Steiner-Tan needle.

In contrast to previous studies, we measured AMH concentrations of all individual follicles within one stimulated IVF cycle of patients undergoing IVF treatment and demonstrated a significant correlation between sAMH and mean/individual fAMH values within patients. This AMH data of all individual mature follicles is unique and supports other studies, which correlated AMH values of the first punctured follicle [20] or pooled FF [21] with sAMH. Peripheral AMH concentrations are exclusively dependent not only on the number of follicles but also on the individual ability of each follicle to produce AMH [22]. Small follicles exhibit higher AMH concentrations compared to large ones, according to the fact that granulosa cells reduce their AMH production during final follicular maturation [23, 24]. Our results also revealed a significant difference in mean fAMH concentrations between patients, which may be based on the varying mature follicle sizes during one stimulated cycle in IVF treatment [25]. Interestingly, mean fAMH showed a trend to be negatively correlated with age, which is in line with the overall AMH decrease in patients with increasing age. Additionally, our results demonstrated decreasing sAMH concentrations with increasing age of the patient, thereby confirming the state-of-the-art opinion that sAMH values inversely correlate with age [7].

In general, fAMH concentrations are positively associated with embryo implantation when measuring the AMH concentrations in the dominant follicle [26]. Studies suggested that higher fAMH values positively correlated with fertilization [27] and implied higher chances for pregnancy [28]. On the other hand, Mehta et al. demonstrated a negative correlation between fAMH and oocyte quality, fertilization, pregnancy, and embryo implantation rates [12]. A major disadvantage of numerous studies is the diverging dataset of FF investigated. It is hardly possible to draw conclusions from pooled FF or dominant follicle analysis only. These data provided hints but did not reflect AMH concentrations in all-grown follicles available with no possibility for future association of individual FF with the respective IVF outcome. Our data provided evidence that fAMH concentrations of individual follicles of one hormonal stimulated cycle during IVF treatment did not significantly differ within one patient and resembled sAMH values.

A limitation of the study is that we did not investigate the respective IVF outcome of different FF and associated embryos. In addition, the small sample size must be considered as a possible drawback. Another limitation is the lack of information on size and volume of the aspirated follicles, since correlation of these parameters with the IVF outcome would lead to a more conclusive analysis. However, volumetric analysis and sizing of follicles are time-consuming procedures and unnecessarily prolong anesthesia and patient discomfort.

## Conclusion

In conclusion, our results clearly showed that individual fAMH concentrations reflected sAMH values of a stimulated cycle during IVF treatment and that fAMH concentrations did not significantly differ within one patient. In future studies, it will be interesting to correlate individual fAMH values to the respective embryo development and overall pregnancy outcome in order to improve IVF treatments and to refrain from embryo overproduction.
